# Does cannabis use in adolescence predict self‐harm or suicide? Results from a Finnish Birth Cohort Study

**DOI:** 10.1111/acps.13384

**Published:** 2021-11-22

**Authors:** Alexander Denissoff, Solja Niemelä, James G. Scott, Caroline L. Salom, Emily Hielscher, Jouko Miettunen, Anni‐Emilia Alakokkare, Antti Mustonen

**Affiliations:** ^1^ Department of Psychiatry Faculty of Medicine University of Turku Turku Finland; ^2^ Addiction Psychiatry Unit Department of Psychiatry Turku University Hospital Turku Finland; ^3^ QIMR Berghofer Medical Research Institute Herston Herston Qld Australia; ^4^ Metro North Mental Health Service Herston Qld Australia; ^5^ Institute for Social Science Research The University of Queensland Brisbane Australia; ^6^ ARC Centre of Excellence for Children & Families over the Life Course The University of Queensland Brisbane Qld Australia; ^7^ School of Public Health The University of Queensland Brisbane Qld Australia; ^8^ Center for Life Course Health Research University of Oulu Oulu Finland; ^9^ Medical Research Center Oulu Oulu University Hospital and University of Oulu Oulu Finland; ^10^ Faculty of Medicine and Health Technology Tampere University Tampere Finland; ^11^ Department of Psychiatry Seinäjoki Central Hospital Seinäjoki Finland

**Keywords:** adolescent, birth cohort, cannabis, self‐harm

## Abstract

**Objective:**

Longitudinal studies examining the association between adolescent cannabis use and self‐harm are rare, heterogeneous and mixed in their conclusions. We study this association utilizing a large general population‐based sample with prospective data.

**Methods:**

The Northern Finland Birth Cohort 1986 (*n* = 6582) with linkage to nationwide register data was used to study the association of self‐reported cannabis use at age 15–16 years and self‐harm and suicide death until age 33 (until year 2018), based on register information. Cox regression analysis with Hazard Ratios (HR) and 95% confidence intervals (CI) was used. Psychiatric disorders, parental psychiatric disorders and other substance use were considered as confounders.

**Results:**

In all, 6582 (49.2% male) were included in the analysis, and 377 adolescents (5.7%) reported any cannabis use until the age of 15–16 years. Based on register information, 79 (55.7% male) had visited in health care services due to self‐harm, and 22 (90.1% male) had died by suicide. In crude analyses, adolescent cannabis use was associated with self‐harm (HR = 3.93; 95% CI 2.24–6.90). The association between cannabis use and self‐harm remained statistically significant after adjusting for sex, psychiatric disorders at baseline, frequent alcohol intoxications, other illicit drug use, and parental psychiatric disorders (HR 2.06; 95% CI 1.07–3.95). In contrast, the association of cannabis use with suicide did not reach statistical significance even in crude analysis (HR 2.60; 95% CI 0.77–8.78).

**Conclusion:**

Cannabis use in adolescence may increase risk of self‐harm independent of adolescent psychopathology and other substance use.

**Significant outcomes**

- Lifetime cannabis use was associated with self‐harm requiring medical attention after adjusting for psychiatric disorders, parental psychiatric disorders, frequent alcohol intoxications and other illicit drug use
- The crude association attenuated by 46% after adjusting for alcohol intoxications and other illicit drug use
- An association between lifetime cannabis use and suicide was not observed

**Limitations**

- Only a dichotomous exposure variable of lifetime cannabis use was used.
- Register‐based outcome data on self‐harm requiring medical attention do not allow to differentiate between suicide attempt and non‐suicidal self‐harm.
- Number of cases with adolescent cannabis use and suicide death was very low, thus introducing power issues.

## 1 INTRODUCTION

Cannabis use has been associated with multiple intentional self‐injury‐related outcomes and a considerable body of evidence has already accumulated regarding the association of cannabis use and subsequent suicide attempt.^1^, ^2^ In contrast, only a handful of longitudinal studies have assessed the association between cannabis use and suicide death with mixed findings.^1^, ^3^, ^4^, ^5^, ^6^ In addition to suicidal behavior‐related outcomes, “self‐harm” and “deliberate self‐harm” defined as intentional self‐injurious behavior regardless of preceding objective have been examined in previous research.^3^, ^7^, ^8^ A recent meta‐analysis of nine cohort studies by Escelsior et al. reported an association between cannabis use and self‐injurious behavior.^9^ Yet, significant knowledge gaps remain in our understanding of the association between specifically adolescent cannabis use and subsequent intentional self‐injury‐related outcomes.

Studying the associations between specifically adolescent cannabis use and different intentional self‐injury‐related outcomes is of paramount importance as adolescence is a time when the brain is most vulnerable and the risk of future psychiatric disorders arising from cannabis use is greatest at this developmental phase.^10^, ^11^ In addition to the indirect effect on self‐harm risk conferred by contributing to the onset of or aggravating preexisting psychiatric disorders, cannabis use has been associated with impulsivity^12^, ^13^ and neurobiological changes putatively of relevance to impulse control.^14^ This is of significance as elevated impulsivity been has associated with intentional self‐injury.^15^ To date, there are 14 prospective longitudinal studies assessing the association between specifically adolescent cannabis use and subsequent suicide attempt (see online Table S3 for more detailed information).^16^, ^17^, ^18^, ^19^, ^20^, ^21^, ^22^, ^23^, ^24^, ^25^, ^26^, ^27^, ^28^, ^29^ However, as far as we know, only one longitudinal study has assessed the association between adolescent cannabis use and subsequent suicide death. There, a negative finding was reported.^3^ Furthermore, there are a paucity of studies assessing the association between adolescent cannabis use and subsequent self‐harm. So far, only three longitudinal studies have assessed this topic^3^, ^7^, ^30^ with two of them reporting^3^, ^7^ significant positive findings (see online Table S2 for more detailed information). However, only one of those studies was general population‐based,^7^ whereas two of them utilized high‐risk samples.^3^, ^30^ The studies were also heterogeneous in terms of lengths of follow‐up and operationalizations of cannabis use and outcome variables. Moreover, while there are high quality birth‐cohort studies assessing the association between adolescent cannabis use and suicide attempt such as Silins et al.,^18^ no studies examining the association of adolescent cannabis use and subsequent severe self‐harm requiring medical attention as a potential indicator of suicide attempt have utilized prospective birth cohort data.

The Northern Finland Birth Cohort (NFBC) 1986 study has comprehensive, general population‐based and prospectively collected data on adolescent lifetime cannabis use, frequent alcohol intoxications, use of other illicit drugs, and baseline and parental psychiatric diagnoses made in clinical practice. These data are linked with nationwide health care registers providing information on diagnostic ICD‐10 codes implying severe intentional self‐harm and data on suicide deaths. Similar outcome measures have been used in previous research.^3^, ^31^ Also, this methodological approach ensures minimal attrition during the follow‐up enhancing the generalizability of the results.

### 1.1 Aims of the study

The aim of our study was to examine the association between lifetime cannabis use at age 15/16 years and self‐harm requiring medical attention and suicide death. We hypothesize that cannabis use is associated with severe self‐harm independent of frequent alcohol intoxication, use of other illicit drugs, sex, and baseline and parental psychiatric disorders. To our knowledge, this is the first study to utilize a birth cohort‐based sample with prospective data and the largest general population‐based study conducted thus far. In addition, the 18‐year follow‐up in our study is longer than in any previous study addressing this issue.

## 2 METHODS

### 2.1 Participants and data‐collection

**FIGURE 1:**
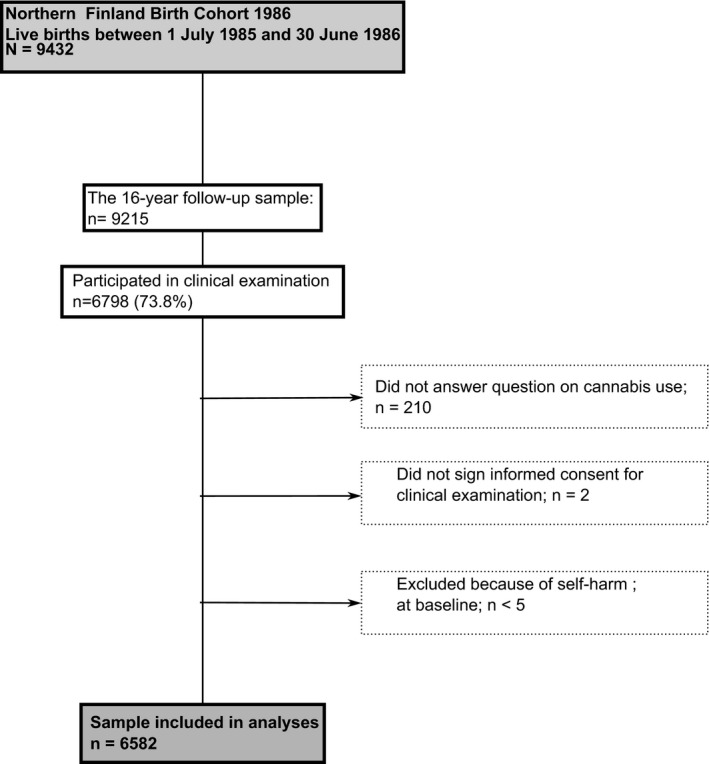
The Northern Finland Birth Cohort 1986

The Northern Finland Birth Cohort 1986 is an ongoing follow‐up study including 99% of all births in the two northernmost provinces in Finland between July 1, 1985 and June 30, 1986. The original sample included 9,432 live born children.^32^ A biphasic follow‐up study was conducted in 2001–2002 when study members were aged 15–16 years. Initially, self‐report postal questionnaires were sent to the adolescents (*n* = 9215) in which they answered questions concerning their health and wellbeing (*n* = 7344). Thereafter, all the participants were invited to a clinical study where they completed self‐report questionnaires including questions on substance use. Participants who provided informed consent, answered questions on cannabis use and did not have a history of self‐harm at baseline were included in the present study. The final sample totaled 6,582 individuals (69.7% of original sample) (Figure 1). The 15‐ to 16‐year follow‐up study was approved by the Ethics committee of the Northern Ostrobothnia Hospital District in Finland (June 14, 1999). The authors assert that all procedures contributing to this work comply with the ethical standards of the relevant national and institutional committees on human experimentation and with the Helsinki Declaration of 1975, as revised in 2008.

#### 2.1.1 Exposure variable: Cannabis use

Data on lifetime adolescent cannabis use were collected during the clinical study when participants were aged 15–16 years. They were asked ‘Have you ever used marihuana or hashish?' as dichotomized (no/yes) and with options ‘never, once, 2–4 times, 5 times or more, or I use regularly'. In the main analyses of this study, lifetime cannabis use was examined as dichotomized (no/yes). Dose‐response was studied post‐hoc for the self‐harm outcome utilizing a four‐class cannabis variable (never, once, 2‐4 times, or 5 times or more). For this analysis, the '5 times or more' and 'I use regularly' groups were pooled due to small sample sizes in the two categories.

#### 2.1.2 Outcome variables: self‐harm and suicide death

Data on suicide‐ and self‐harm‐related diagnostic codes (ICD‐10: X60‐X84, Z91.5, Y87.0 and Z72.8 as well as ICPC‐2: P77) were collected cumulatively from the participants´ age of 16 years until the end of 2018, when participants were aged 33 years, that is, follow‐up was altogether 18 years. Data on these diagnostic codes were obtained from the Care Register for Health Care 2001–2018 and the Register of Primary Health Care Visits 2011 – 2018. The Care Register contains information on patients discharged from inpatient care, and since 1998 also on specialized outpatient care. The Register of Primary Health Care Visits includes all outpatient primary health care delivered in Finland. For more information concerning, these registers see previous studies.^31^, ^33^, ^34^

Information on suicides as causes of death until the end of 2018, that is, until age 33 years, was obtained from the Registry for Causes of Death which covers all deaths in Finland. An autopsy is routinely carried out in all unclear cases in Finland. Deaths are categorized based on cause of death according to the ICD‐10 (WHO 2016) and categorized into deaths due to somatic causes, accidents, and suicide. Information on non‐suicide deaths and times of emigration from Finland, which were used as censoring points in our analyses, were obtained from the Population register data and the Registry for causes of death.

### 2.2 Alcohol use and other illicit substance use

Data on lifetime illicit substance and frequent alcohol intoxications use were collected at age 15–16 years using a questionnaire during the clinical study. The participants were asked: ‘Have you used ecstasy, heroin, cocaine, amphetamine, LSD or other similar intoxicating drugs?’ A person was categorized to the ‘yes’‐group if person had used any of these substances at least once. Frequent alcohol intoxications were questioned as ‘Have you been drunk during the past year? (0, 1–2, 3–5, 6–9, 10–19, 20–39, or 40 times or more)’, and this was categorized as ‘Have you been drunk 10 times or more during the past year?—(no/yes)’.

### 2.3 Psychiatric disorders before age 16 years and parental psychiatric disorders

Data on psychiatric diagnostic codes (ICD‐10: F00‐F69, F80‐F99) recorded before the participants were aged 16 years were obtained from the following registers until 2002: The Care Register for Health Care, the medication reimbursement register of the Social Insurance Institution of Finland, and the disability pensions of the Finnish Center for Pensions. The Care Register contains information on patients discharged from inpatient care, and since 1998 also on specialized outpatient care.

Data on lifetime parental psychiatric diagnoses were obtained from the nationwide Registers of Health Care during the years 1972—2018 (includes inpatient care and visits to specialized outpatient health care since 1998, and primary health care since 2011), and Finnish Center for Pensions until 2016. The variable was classified dichotomously as whether either parent had been diagnosed with an ICD‐10 psychiatric disorder.

### 2.4 Statistical analyses

We used crosstabulation and chi‐squared or Fisher's exact test, as appropriate, to assess the relationship of lifetime cannabis use, self‐harm, and suicide. Linear regression and multicollinearity diagnostics with variance inflation factor (VIF) scores were used to detect correlation between multiple covariates. VIF >5 was used as an indicator of multicollinearity.

We applied Cox regression analysis with hazard ratios (HR) and 95% confidence intervals (CI) to study the association of adolescent cannabis use with subsequent self‐harm and suicide. Times at emigration from Finland (*n* = 265) and non‐suicide‐related deaths (*n* = 28) were used as censoring points in survival analyses. The Cox proportional hazard assumption was examined by using hazard logarithms, scaled Schoenfeld residuals, and time‐dependent covariates. The models are as follows: Model 1: sex and psychiatric diagnosis at baseline. Model 2: sex, psychiatric diagnosis at baseline, frequent alcohol intoxications past 12 months, lifetime use of other illicit drugs than cannabis. Model 3: sex, psychiatric diagnosis at baseline, frequent alcohol intoxications past 12 months, lifetime use of other illicit drugs than cannabis and parental psychiatric disorders. Cox regression analysis was also used to study possible interactions between lifetime cannabis use and all covariates included in Model 3. The Aalen‐Johansen cumulative incidence curve was computed for the self‐harm outcome. We also studied dose‐response for self‐harm with a trend test, in which cannabis use was studied as a continuous variable in Cox regression analysis utilizing the fully adjusted model (Model 3).

Previous attrition analyses of this sample have shown that fewer males (64% vs. 71%; *p*<0.001), individuals living in urban areas (66% vs. 71%, *p*<0.001) and individuals with parental psychiatric disorder (58% vs. 69%, *p*<0.001) participated in the 15–16 year follow‐up study (Miettunen et al. 2014). Thus, inverse probability weighting^35^ was used to weight the sample data by sex, parental psychiatric disorder and urbanicity. Both the weighted and the unweighted data were analyzed with logistic regression analysis and odds ratios (OR). Statistical significance was retained in the weighted analyses of cannabis use and incident self‐harm for all those associations that were statistically significant in the unweighted analyses, and the strength of the associations was of similar magnitude (data available from the authors). Also, to study the effects of nonresponse, descriptive statistics on covariates and cannabis use were tabulated for the crude and final models.

Statistical analyses were performed using SPSS statistical software (IBM SPSS Statistics, version 25; IBM Co., Armonk, New York, USA) except for Aalen—Johansen hazard curves, and examination of Cox proportional hazard assumption that were analyzed using the R programming environment (R version 3.6.0, R foundation for statistical computing, Vienna, Austria).

## 3 RESULTS

**FIGURE 2:**
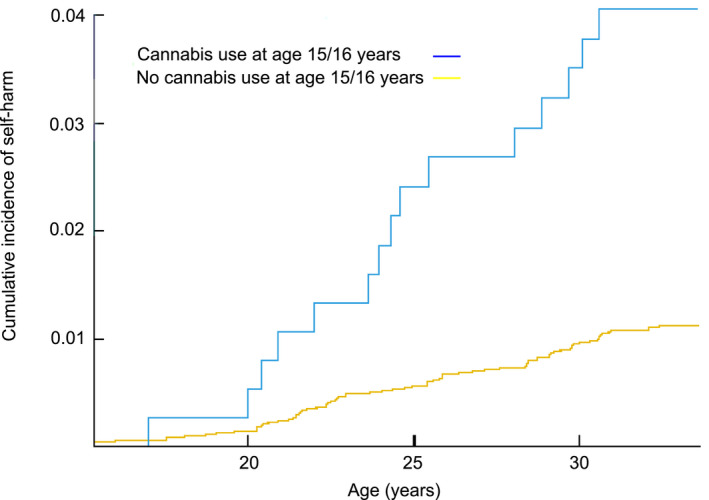
The Aalen‐Johansen Cumulative Incidence Curve for the Self‐Harm Outcome

The variables under study and their relation to lifetime cannabis use and self‐harm and suicide during the follow‐up are presented in Tables 1 and 2. The sample totalled 6582 individuals, of which 377 adolescents (5.7%) reported any cannabis use until the age of 15–16 years. In our data set, 311 (4.7%) adolescents reported having tried cannabis 1–4 times and 66 (1.0%) 5 times or more frequently. Girls reported any cannabis use more commonly than boys (56.0% v 44.0%, *p* = 0.038). For more comprehensive data regarding the distribution of cannabis use in this sample, please see Table 1. Significant multicollinearity was not seen (all VIFs <1.2). Based on register information 1.2% had an ICD‐10 diagnosis code based on a self‐harm incident (n= 79/6582, 56% male) and 0.3% died by suicide (n=22/6582, 91% male) suicide. Less than five individuals both received an ICD‐10 diagnosis implying severe self‐harm and died by suicide. A dose‐response for the self‐harm outcome was seen with a trend test (HR = 1.87; 95% CI 1.17–3.00). The Aalen‐Johansen curve for cumulative incidence of self‐harm by cannabis use status is presented in Figure 2.

**TABLE 1:** Association of covariates and cannabis use in Northern Finland Birth Cohort 1986

				Cannabis use frequency				
	Total n	*n* = 6582		No cannabis use *n* = 6205		Once or more *n* = 377		*p*‐value
		n	%	n	%	n	%	
Sex								
Male	6582	3239	49.2	3073	49.5	166	44.0	0.038
Female		3343	50.8	3132	50.5	211	56.0	
Psychiatric disorder at baseline								
No	6582	6325	96.1	5973	96.3	352	93.4	0.005
Yes		257	3.9	232	3.7	25	6.6	
Cannabis use								
No	6582	6205	94.3	6205	100.0	0	0.0	–
Yes		377	5.7		0.0	377	100.0	
Other illicit drug use								
No	6554	6519	99.5	6171	99.9	348	92.6	<0.001*
Yes		35	0.5	7	0.1	28	7.4	
Alcohol intoxication 10 ≥ times past year								
No	6419	5227	81.4	5105	84.4	122	32.8	<0.001
Yes		1192	18.6	942	15.6	250	67.2	
Parental psychiatric disorder								
No	6582	4174	63.4	3949	63.6	225	59.7	0.123*
Yes		2408	36.6	2256	36.4	152	40.3	

* Fischer´s exact test.

**TABLE 2:** Association of covariates, self‐harm and suicide death in Northern Finland Birth Cohort 1986

		Self‐harm					Suicide death				
	Total *n*	No *n=6503*		Yes *n=79*		*p*‐value	No *n* = 6560		Yes *n* = 22		*p*‐value
		n	%	n	%		n	%	n	%	
Sex											
Male	6582	3195	49.1	44	55.7	0.259*	3219	49.1	20	90.9	<0.001
Female		3308	50.9	35	44.3		3341	50.9	2	9.1	
Psychiatric disorder at baseline											
No	6582	6261	96.3	64	81.0	<0.001*	6304	96.1	21	95.5	0.584*
Yes		242	3.7	15	19.0		256	3.9	1	4.5	
Cannabis use											
No	6582	6141	94.4	64	81.0	<0.001*	6186	94.3	19	86.4	0.128*
Yes		362	5.6	15	19.0		374	5.7	3	13.6	
Other illicit drug use											
No	6554	6443	99.5	76	96.2	0.008*	6497	99.5	22	100.0	1.00*
Yes		32	0.5	3	3.8		35	0.5	0	0.0	
Alcohol intoxication 10 ≥ times past year											
No	6419	5183	81.7	44	57.1	<0.001	5213	81.5	14	66.7	0.092*
Yes		1159	18.3	33	42.9		1185	18.5	7	33.3	
Parental psychiatric disorder											
No	6582	4147	63.8	27	34.2	<0.001	4166	63.5	8	36.4	0.008
Yes		2356	36.2	52	65.8		2394	36.5	14	63.6	

* Fischer´s exact test.

The association of cannabis use with suicide did not reach statistical significance even in crude analysis (HR 2.60; 95% CI 0.77–8.78). The results of the multivariable analyses for cannabis use and self‐harm are summarized in Table 3. Regarding sex, time‐dependency was seen both examining the hazard logarithm curves and utilizing the time‐dependent covariate. Violations of the proportionality of hazards assumption were not detected concerning cannabis use or other covariates included in the final model. A crude association of adolescent cannabis use, and risk of self‐harm was seen. When adjusted for sex and psychiatric disorders at baseline, the association between cannabis use and subsequent self‐harm attenuated but remained statistically significant (HR =3.75; 95% CI 2.13–6.61). After adjusting for frequent alcohol intoxications and illicit drugs other than cannabis, the association attenuated by 46 percent (HR 2.04; 95% CI 1.07–3.90). This association remained statistically significant after further adjusting for parental psychiatric disorders (HR 2.06; 95% CI 1.07–3.95). Stratified analyses did not reveal any statistically significant interactions between cannabis use and any covariates included in the final model (sex, psychiatric disorder at baseline, frequent alcohol intoxications, use of other illicit drugs and parental psychiatric disorders, Model 3). Descriptive statistics on the effects of nonresponse on sample characteristics indicate, that the participants included in the final model (Model 3) did not differ substantially from the whole (Crude model) sample in terms of cannabis use or any covariate included (see online Table S1 for more detailed information).

**TABLE 3:** The hazard ratios (HR) for the risk of self‐harm in Northern Finland Birth Cohort 1986 by cannabis use status

		Sample size	No cannabis use at baseline	Cannabis use at baseline		
			Self‐harm	Self‐harm	HR	95% CI
Self‐harm	Crude	6576	64	15	**3.93**	2.24–6.90
	Model 1	6576	64	15	**3.75**	2.13–6.61
	Model 2	6388	62	15	**2.04**	1.07–3.90
	Model 3	6388	62	15	**2.06**	1.07–3.95

Note

Model 1: sex, psychiatric disorder at baseline; Model 2: sex, psychiatric disorder at baseline, frequent alcohol intoxications past year, use of other illicit drugs; Model 3: sex, psychiatric disorder at baseline, frequent alcohol intoxications past year, use of other illicit drugs, parental psychiatric disorder. Statistically significant results in **bold**.

## 4 DISCUSSION

In this large general population‐based prospective study with an 18‐year follow‐up, we report an association between adolescent cannabis use and subsequent self‐harm requiring medical attention. This association remained statistically significant after adjusting for sex, baseline and parental psychiatric disorders, frequent alcohol intoxications and use of other illicit drugs than cannabis at age 15/16 years. However, an association was not seen between adolescent cannabis use and suicide death. To our knowledge, this is the largest study examining these associations using prospective, general population‐based data.

We found that association between cannabis use and self‐harm attenuated by 46% after adjusting for frequent alcohol intoxications and other illicit drug use. This is in line with previous research, as associations between high‐risk alcohol use in adolescence and self‐harm have been reported.^7^ Also, heavy acute alcohol use is strongly associated with specifically subsequent suicide attempt.^36^ Furthermore, polysubstance use in adolescence is common and introduces a potential source of confounding.^37^

In all, the previously published longitudinal studies assessing the association between adolescent cannabis use and self‐harm have been quite heterogeneous in terms of measurement of self‐harm, sample sizes and characteristics (see Table S2 for more detailed information). Moran et al utilized data from the Victoria Adolescent Health Cohort, a population‐based stratified random sample drawn from 44 schools.^7^ In this study self‐harm was defined as encompassing even “deliberate non‐recreational risk taking”. The two other longitudinal studies were conducted with cohorts of high‐risk individuals. Fontanella et al. utilized a cohort of mood disorder patients with comorbid cannabis use disorder (CUD) as the exposure of interest.^3^ Spears et al. utilized a sample of socio‐economically deprived adolescents.^30^ Furthermore, the data used in this study was intended for a randomized clinical trial.^30^

The cumulative incidence of self‐harm operationalized as ICD‐10 diagnoses made in clinical practice was low in this study (1.2%). Studies utilizing self‐reported self‐harm as an outcome have uniformly reported higher outcome incidences.^7^, ^30^ However, our outcome was based on self‐harm related health care visits, and thus it is possible that this data reflects incidents related to more severe self‐harm than compared to a self‐report measure. As in our study, Fontanella et al. operationalized self‐harm as ICD‐10 diagnoses made in clinical practice and thus reported a cumulative incidence of only 1.0%.^3^ In line with our findings, a significant positive finding was reported. However, whereas lifetime use was the main exposure variable in our study, Fontanella et al. focused on the association between cannabis use disorder (CUD) and self‐harm.

In this study, the association between cannabis use and suicide was statistically nonsignificant even in crude analysis. Fontanella et al. 2021 reported a crude association between CUD and suicide, which attenuated to statistically nonsignificant in their final model^3^ despite the high‐risk nature of their sample constituting of mood disorder patients including those with prior history of self‐harm, formidable sample size (*n* = 204780) and a robust exposure variable (CUD). However, due to the short follow‐up period of one year, only 30 suicide deaths were captured. As noted earlier, longitudinal studies conducted on adult populations concerning this association have also yielded equivocal findings.^4^, ^5^, ^6^ This is probably due to methodological differences pertaining to operationalizations exposure variables and covariates selected in statistical models.

The strengths of this study are as follows: The NFBC1986 birth cohort provides data on a large community sample with high ethnic and genetic homogeneity. As far as we know this is the second prospective general population‐based study addressing this issue, the first one to use birth‐cohort data and the longest in terms of follow‐up duration. To our knowledge, no other studies with prospective data have examined the association between adolescent cannabis use and severe self‐harm requiring medical attention by utilizing a linkage to nationwide registers. Also, assessing register‐based parental psychiatric diagnoses made in clinical practice is a unique feature of this study. Additionally, there is almost complete participant retention with only a very small proportion of cohort members deceased or emigrated during the follow‐up.

However, there are also limitations. The outcomes were rare (1.2% for self‐harm and 0.3% for suicide death) and only 5.7% reported using any cannabis lifetime use at age 15/16 years in this sample introducing power issues and increasing the likelihood of type II error. In the ESPAD survey conducted in 2003, the lifetime prevalence of cannabis use at the age of 15/16 in Finland was 11%, which suggests that underreporting might be an issue with our data.^38^ However, this source of bias would more likely weaken the associations observed rather than to inflate them, so we may be confident of our finding. Furthermore, use of a multi‐class cannabis use frequency variable in the multivariate models was not possible due the low number of cases. However, similar cannabis use variables have been utilized in previous prospective longitudinal studies addressing this topic.^7^, ^30^ Also, as this study focused on incident self‐harm requiring medical attention, we chose to exclude the few subjects who presented with self‐harm at baseline (n < 5) from the final sample. However, not having more detailed information on self‐harm and suicidal behaviors can be considered as a limitation. For instance, when using register‐based information, one cannot differentiate between non‐suicidal self‐injury and suicide attempt. Also, utilizing register‐based information rather than self‐report of self‐harm may lead to underreporting. Furthermore, we were not able to control for the potentially confounding effect of parental self‐harm or suicide death. However, utilizing nationwide health care register data, we could capture the most severe self‐harm‐related incidents with no attrition during an 18‐year follow‐up.

Adolescent cannabis use associated with risk of subsequent severe self‐harm. In our study, this association was observed after extensive confounder control, that is, independent of sex, baseline and parental psychiatric disorders, frequent alcohol intoxications and other illicit drug use. There are a paucity of longitudinal studies on adolescent cannabis use and subsequent severe self‐harm and death by suicide. A large general population‐based study with a repeated‐measures design and a multi‐class cannabis exposure variable reflecting intensity of use would shed further light on this issue.

## AUTHOR CONTRIBUTIONS

AD, AM, SN, and AEA had full access to all the data in the study and takes responsibility for the integrity of the data and the accuracy of the data analysis. AD, AM, and SN developed conception and design of the work. AD, AM, SN, AEA, JM, CS, EH, and JGS performed data analysis and interpretation and wrote the first manuscript draft. AD, AM, SN, AEA, JM, CS, EH, and JGS supervised conception and design of the work and provided critical revision of the article. All authors contributed approval of the final version of the manuscript.

## ROLE OF THE FUNDING SOURCE

The funders had no role in the design and conduct of the study; collection, management, analysis, and interpretation of the data; preparation, review, or approval of the manuscript; and decision to submit the manuscript for publication.

### PEER REVIEW

The peer review history for this article is available at https://publons.com/publon/10.1111/acps.13384.

## Supporting information

Supplementary Material

## Data Availability

The data that support the findings of this study are available from University of Oulu. Restrictions apply to the availability of these data, which were used under license for this study. Data are available from the author(s) with the permission of University of Oulu.
