# Supplementary material for: Does cannabis use in adolescence predict self‐harm or suicide? Results from a Finnish Birth Cohort Study
Source: Acta Psychiatr Scand. 2021 Nov 22;145(3):234–43. doi: 10.1111/acps.13384 (PMC9299098; doi:10.1111/acps.13384)
Supplement: Supplementary file 1 — Supplementary Material [file ACPS-145-234-s001.docx]

Online supplement table 1. Frequencies and percentages of covariates of within different cannabis use categories

|  | Crude model | |  |  | Model 3 | |  | |
| --- | --- | --- | --- | --- | --- | --- | --- | --- |
|  | No use | Use at baseline |  |  | No use | Use at baseline | |  |
| **Sex** |  |  |  |  |  |  | |  |
| Male | 3073 | 166 |  |  | 2955 | 165 | |  |
|  | *49.5%* | *44.0%* |  |  | *49.1%* | *44.5%* | |  |
| Female | 3132 | 211 |  |  | 3062 | 206 | |  |
|  | *50.5%* | *56.0%* |  |  | *50.9%* | *55.5%* | |  |
| **Psychiatric diagnosis at baseline** |  |  |  |  |  |  | |  |
| No | 5973 | 352 |  |  | 5792 | 346 | |  |
|  | *96.3%* | *93.4%* |  |  | *96.3%* | *93.3%* | |  |
| Yes | 232 | 25 |  |  | 225 | 25 | |  |
|  | *3.7%* | *6.6%* |  |  | *3.7%* | *6.7%* | |  |
| **Other illicit drug use** |  |  |  |  |  |  | |  |
| No | 6171 | 348 |  |  | 6010 | 343 | |  |
|  | *99.9%* | *92.6%* |  |  | *99.9%* | *92.5%* | |  |
| Yes | 7 | 28 |  |  | 7 | 28 | |  |
|  | *0.1%* | *7.4%* |  |  | *0.1%* | *7.5%* | |  |
| **Alcohol intoxication 10 ≤ times past year** |  |  |  |  |  |  | |  |
| No | 5105 | 122 |  |  | 5078 | 122 | |  |
|  | *84.4%* | *32.8%* |  |  | *84.4%* | *32.9%* | |  |
| Yes | 942 | 250 |  |  | 939 | 249 | |  |
|  | *15.6%* | *67.2%* |  |  | *15.6%* | *67.1%* | |  |
| **Parental psychiatric disorder** |  |  |  |  |  |  | |  |
| No | 3949 | 255 |  |  | 3824 | 221 | |  |
|  | *63.6%* | *59.7%* |  |  | *63.6%* | *59.6%* | |  |
| Yes | 2256 | 152 |  |  | 2193 | 150 | |  |
|  | *36.4%* | *40.3%* |  |  | *36.4%* | *40.4%* | |  |
| **Self-harm**  No | 6141 | *362* |  |  | *5955* | *356* | |  |
|  | *99.0%* | *96.0%* |  |  | *99.0%* | *96.0%* | |  |
| Yes | *64* | *15* |  |  | *62* | *15* | |  |
|  | *1.0%* | *4.0%* |  |  | *1.0%* | *4.0%* | |  |

Model 3: sex, psychiatric disorder at baseline, other illicit drug use, frequent alcohol intoxications, parental psychiatric disorder

Online supplement table 2: Longitudinal studies assessing the association between adolescent cannabis use and subsequent self-harm

| Study (year) | Sample | Sample size | Follow up (years) | Predictor | Outcome (n, %) | Assessment measure | Point estimate | 95 % Confidence intervals | Covariates |
| --- | --- | --- | --- | --- | --- | --- | --- | --- | --- |
| Moran et al. (2012) | Australia - Population based, 14/15 years | 1802 | 15 years at most | Any use during past 6 months | Including risk taking  Incident SH adolescence  8.3 %  Incident SH  young adult  2.6 % | Interview | **HR 1.8**  OR 1.8 | 1.0–3.1  0.45-7.6 | Sex, Family structure, depression or anxiety, **high-risk alcohol use, cigarette smoking** |
| Spears et al. (2014) | Chile – adolescents, low socioeconomic bacground | 1,582 (77 %) participants reporting  no SH at baseline | 0.5 | Exposure (never/at least once) | At 6-month follow-up, 14 % (n = 220/1,582) of participants  with no reported SH at baseline reported incident SH. | Interview | aOR 1.43 | 0.83-2.49 | BDI (low/high), anxiety, school connectedness, rational problem solving, suicidal thoughts, **alcohol, smoking** |
| Fontanella  (2021) | US Medicaid data  204 780  Mood disorder patients  Mean age 17.2  1.0 % prior self-harm | 204 780 | 1 | CUD (ICD-10) | Non-fatal SH  2126/204780  1.0 %  Suicide  30/204780 | Register-based ICD-10 diagnosis | **aHR**  **3.28**  1.22 | 2.55-4.22  0.44-3.43 | Age, sex, ethnicity, health insurance status, recidence, psychiatric comorbidities (**incl. SUD**), prior psychiatric history |

Statistically significant findings and substance use covariates in **bold**

Online supplement table 3: Prospective longitudinal studies assessing association between adolescent cannabis use and suicide attempt

| Study (year) | Sample | Base  line  age | Sample size | Follow up (years) | Predictor | Attempt  (n, %) | Assessment measure | Point estimate | 95 % Confidence intervals | Covariates adjusted for |  |
| --- | --- | --- | --- | --- | --- | --- | --- | --- | --- | --- | --- |
| Agrawal et al. (2017) | US-COGA, high genetic risk for AUD:s | 22-26  y/o  (1.3 % > 22  y/0) | 3277 | 10 | Cannabis use before age of 15 yrs | 190, 5.8 % | SSAGA | aOR 1.13 | 0.76-1.67 | gender, Hispanic ethnicity, parental AUD, MDD, externalizing disorders |  |
| Borowski et al. (2001) | US – Add Health | Grades  7. – 12. | 13110 | 0.92 | Cannabis use | 474, 3.6 % | “Interview” | aORs  G. black **10.30**  G. hisp. **4.50**  G. whites **3.40** B. black  **5.90**  B. hisp. **2.90**  B. whites **6.80** | Not reported  in publication  due to space constraints | Age, family structure, welfare status, ethnicity |  |
| Borges et al. (2017) | Mexico-  MAMHS | 12-17  y/o | 1071 | 8 | Past 12-mo use:  < 1x/mo  > 1-3/mo  DSM-IV DUD within 12 mo | 61, 5.9 % | WMH-CIDI | aRR:s  1.64  **4.60**  **4.74** | 0.17.16.08  1.03-20.60  1.09-20.57 | Gender, age/cohort, education, attending school, any DSM-IV anxiety disorders, any DSM-IV mood disorders, | any DSM-IV  impulse disorders and any DSM-IV eating disorders. |
| Clarke et al. (2014) | Dublin, Ireland | 12-15 y/o | 168 | Approx. 7 | Ever used at 12-15 y/o | 17, 10.1 % | “Interview” | aOR **7.50** | 1.20-43.80 | Family psychiatric history, childhood trauma, | **alcohol** and other psychopathology |
| Hengartner et al. (2020) | Zurich, Switzerland | 19-20  y/o | 591 | 30 | Use < 20 y/o | “suicidality” | SPIKE | aoR **1.64** | 1.09-2.48 | assessment year , sex, family climate, social support, | parental income, education level **drug abuse, and alcohol abuse** |
| Juon & Ensminger (1997) | US – Chicago, IL | 15-16  y/o | 953 | Approx 17 | Lifetime use more/less than 40 x | 39, 4.1 % | “Interview” | OR  Male  0.34  Female 1.84 | 0.04-2.85  0.50-6.82 | Crude only |  |
| Mars et al. (2019) | UK – ALSPAC | 16  y/o | 456 (thoughts at BL)  569  (parasuicidality at BL) | 5 | At least occasional use | 38/310, 12 %  46/380,  12 % | questionnaire | aOR  **th. 2.61**  **ps. 2.14** | 1.11-6.14  1.04-4.41 | gender, socioeconomic position |  |
| Pedersen et al. (2008) | Norway -Young in Norway | 12-16  y/o &  21 y/o | 2033 | 13 &  6 | Early use  21 y/o use  1-10 x  11 + x | 229, 1.1 % | questionnaire | N/A  aOR  0.7  **2.9** | 0.4-1.5  1.3-61 | age, gender, parental educational level, parents unemployed or  receiving social welfare benefits, parental divorce, parental smoking and alcohol  problems, parental support and monitoring measured at the age of 16 years, | early  pubertal maturation, school marks, conduct problems and **daily**  **smoking**, **alcohol intoxication**, **alcohol**  **problems**, depression, impulsivity, level of education, unemployment and income from social  security, marriage ⁄ cohabitation and being a parent. |
| Rasic et al. (2013) | Canada – Nova Scotia, AHS | Grade 10. | 976 | 2 | Past 30 d:  > 10 x  3-9 x  1-2x | 35, 3.6 % | survey | aOR  1.04  1.03  1.01 | 0.98-1.10  0.99-1.07  0.98-1.05 | time dynamic school mark, living arrangement, | **alcohol, drug use** and depression |
| Roberts et al. (2010) | US – Houston, Tx, TH2K | 11-17  y/o | 3134 | 1 | Use in last year | All 0.95 %, 1^st^ att. 0.84 % | DISC-IV | aOR 4.70 | Not reported | Age, gender,family ncome,prior suicide attempts by youths and by their caregivers, | pre-existing youth psychiatric disorders,personal and social resources,and lifestress. |
| Silins et al. (2014) | New Zealand Australia – CHDS,  VAHCS | < 17 y/o | 2537 | Not available | < 17 y/o:  < monthly  monthly +  weekly +  daily | 78, 3.1 % | survey | aOR  **1.62**  **2.61**  **4.23**  **6.83** | 1.19-2.19  1.43-4.79  1.71-10.47  2.04-22.90 | Age, sex, baseline  depression, **alcohol use,**  **smoking** |  |
| Thompson & Light (2011) | US – NLSAH | Grades  7.-12. | 10828 | 1 &  7 | Marijuana use | Not reported | survey | aOR  1.03  N/A | 0.97–1.10 | All factors reaching significance in bivariate | analyses + gender  nteractions |
| Weeks & Colman (2017) | Canada – NLCSY | 14-15  y/o | 6788 | 2 | Marijuana/ cannabis at least  1-2 x/mo | 291/6788, 4.3 % | interview | aOR  **1.87** | 1.09-3.22 | Depression at baseline,  SES, family dysfunction, stressful life event, | chronic disease in child,  behavior problems |
| Wilcox & Anthony (2004) | US,  Mid-Atlantic  region | 8-15 | 2311 | Approx.  15 | Use before 15 y/o | 155/1695, 9.1 % | interview | aRR  Both  **1.8**  Male  2.30  Females  2.10 | 1.0–3.3  0.70-7.50  0.90-4.70 | Ethnicity, MDD at baseline, **alcohol, tobacco, illicit drugs,** | aggression, parental psychiatric disturbance, deviant peers |

Statistically significant findings and substance use covariates in **bold**
